# Crisis leadership in COVID-19: A qualitative study of Norwegian business leaders

**DOI:** 10.3389/fpsyg.2022.937935

**Published:** 2022-08-22

**Authors:** Vibeke Dale Oen, Jeanett Svihus, Sara Helene Røyland Solberg, Anette Harris, Jarle Eid

**Affiliations:** Department of Psychosocial Science, University of Bergen, Bergen, Norway

**Keywords:** crisis leadership, pandemic (COVID-19), positive outcome expectancies, resilience, coping, sensemaking

## Abstract

**Background:**

On March 11, 2020, the World Health Organization declared the novel coronavirus outbreak a global pandemic. The crisis that follows presented significant adverse challenges for organizations and business leaders around the world. The present study aims to explore how the extreme context of the COVID-19 influenced crisis leadership, with emphasis on coping and adaptive approaches, in Norwegian leaders during the early stage of the pandemic.

**Materials and methods:**

A group of 11 Norwegian business leaders from different private sector companies were subject to an in depth, semi structured interview after the first 9 months of COVID-19. A sensemaking perspective and the Cognitive Activation Theory of Stress (CATS) were used to interpret the results.

**Results:**

The pandemic called for crisis leadership and a rapid adaptation to a radically changed situation. Restructuring of organizational processes and introduction of new routines were followed by support and caring for their employees during the first wave of the pandemic. All the leaders coped well with the situation, and some were excited over the opportunity to make a difference in this demanding and stressful situation. Many emphasized that the pandemic was an external threat, resulting in an acceptance of the situation, more transparency, collaboration, and generosity within the organization. Especially the willingness to change was challenged in a positive way. A more blurred line between office and home, and absence of social activities were mentioned as negative outcomes.

## Introduction

In March 2020, the COVID-19 virus turned into a global pandemic with significant adverse consequences for organizations and leaders (Garretsen et al., 2022). Unlike leadership challenges in response to previous extreme events such as the Bhopal Chemical release (Bowman and Kunreuther, 1988), Three Mile Island meltdown (Hopkins, 2001), or the Tenerife airplane collision (Weick, 1990) the pandemic presented an extreme context that had a prolonged impact on leadership in public (McLeod and Dulsky, 2021) and private (Garretsen et al., 2022) sector organizations. Leadership requires careful adaptation to the context and the situation (Yukl et al., 2002) and leadership effectiveness is in large part dependent on successful adaptation to salient contextual factors (Osborn et al., 2002). Thus, the pandemic presented an opportunity to study “*leadership in adversity*” by exploring how business leaders applied *crisis leadership* in response to the *extreme context* of COVID-19. According to Hannah et al. (2009) “an *extreme context* is an environment where one or more extreme events are occurring or are likely to occur that may exceed the organization’s capacity to prevent and result in an extensive and intolerable magnitude of physical, psychological, or material consequences to—or in close physical or psycho-social proximity to—organization members,” (p. 898).

COVID-19 and subsequent measures to control the spread of the virus presented an extreme context for many business enterprises (Garretsen et al., 2022). The pandemic produced an extreme context, in that it; (1) had the potential for massive physical, psychological, or material consequences for the employees and organization, (2) the consequences in the form of lay-offs foreclosures, or bankruptcy would have significant consequences for customers and employees, and (3) the external events exceed the organization’s capacity to prevent the extreme events from taking place (adapted from Hannah et al., 2009, p. 898). From an interactional person-by-situation paradigm the pandemic therefore provided an opportunity to explore crisis leadership as embedded in—and socially constructed from an extreme and adverse context as the events unfolded (Larsson and Eid, 2012).

A pressing issue in crisis leadership is to understand how a crisis is interpreted by leaders and subsequently how leaders exert influence on the affect, cognitions, and behaviors of employees and significant stakeholders (Bundy et al., 2017). Faced with the personal risk from the pandemic, business leaders had to adopt holistic and flexible strategies to manage the unexpected and potentially disruptive effects of the pandemic (Garretsen et al., 2022). The uncertainties associated with the cascading effects of COVID-19 posed a significant disruption that called for a swift response and a radical shift from risk management to crisis leadership (Pescaroli et al., 2021). Strict measures to contain the virus such as lockdown, quarantine, travel restrictions, or isolation had a severe impact on employees and most business sectors. Still, an adverse situation such as the pandemic can also become a turning point for positive change, personal growth, or even new business opportunities if the crisis is well managed (James et al., 2011). In their recent review of crisis leadership studies Wu et al. (2021) noted that while studies of cognitive and behavioral aspects of crisis leadership was common, the emotion management process through which leaders can mitigate the negative emotions and restore the positive emotions of stakeholders during crises is less understood. The present study contributes to this end by exploring how a group of Norwegian business leaders responded in the early stages of the pandemic. More specifically, our study seeks to answer the following research question:

*How did the extreme context of the COVID-19 influence the crisis leadership of Norwegian business leaders in the early stage of the pandemic?*

### Relevant literature

While the topic of crisis leadership has been subject to multiple studies over the last decades, research in this field remains fragmented according to the most recent review by Wu et al. (2021). Crises can generally be understood as unexpected and highly salient events that are perceived by leaders and organizational stakeholders as adverse and unwanted (Bundy et al., 2017). It follows from this that although crises are relatively rare, they pose a significant risk due to their severe consequences and the urgent need for leaders and organizations to implement preventive measures. Effective crisis leadership is therefore important for the survival and growth of the organization. Crisis leadership can be defined as *“a process in which leaders act to prepare for the occurrence of unexpected crises, deal with the salient implications of crises, and grow from the disruptive experience of crises,”* (Wu et al., 2021, p. 2). Since leader effectiveness is dependent upon the context (Osborn et al., 2002), effective crisis leadership will depend on how the leader adapts to the adverse and salient aspects of the crisis. So, in this case, what emerged as the most salient contextual aspects from COVID-19 that the business leaders had to attend to?

According to Hannah et al. (2009, p. 902–908), several dimensions of extreme contexts will have a crucial impact on crisis leadership. (1) The location in time or the temporal ordering of the adverse events will present a challenge. The rapid spread of the virus and fluctuations in the symptom load over time made it difficult for leaders in the public and private sector to plan and prepare for normalcy. (2) The potential magnitude of consequences from the threat and the probability of the consequence occurring would present another issue. The adverse health risks for leaders and employees could be significant, particularly in the first phase of the pandemic before vaccines were available. (3) Physical, psychological, or social proximity to the disruptive events represents another dimension of an extreme context. The nature and spread of the virus, including incubation time, made it very difficult for business leaders and employees to protect from the virus by avoiding contact with customers or clients. (4) Finally, Hannah et al. (2009) points out that the form of threat is a salient aspect of an extreme context. Faced with COVID-19, the virus was an invisible and potentially deadly agent with a high potential to stoke anxiety and fear of contamination and disease in employees, their family, and friends.

Faced with these kinds of extreme situations, leaders are clearly important to come to terms with and search for answers to make sense of what is happening (Weick et al., 2005). Sensemaking is considered a key task for leaders in crisis situations in that they develop a shared mental model of how to assess, understand, and respond to the crisis (Weick, 1988). Still, the cognitive diversity and consensus in leaders who are exposed to the same contextual events may reveal significant differences and similarities in their beliefs and between their mental models (Combe and Carrington, 2015). In a recent review of organizational sensemaking Christofaro (2022) offers an updated and holistic revisitation of the original sensemaking model and proposes a co-evolutionary framework of organizational sensemaking emphasizing the significance of emotional schemata in collective sensemaking processes. Not surprisingly, sensemaking have been applied to investigate the emotionally charged aspects of organizational and leadership processes during the pandemic (Stephens et al., 2020; Christianson and Barton, 2021). The emotional aspects of collective sensemaking processes are particularly relevant in crisis leadership situations where the organization are facing extreme contextual factors (Medeiros et al., 2022).

### Theoretical framing

Emerging research on crisis leadership and organizational change following COVID-19 suggest the emergence of new work-related challenges, but also points to new opportunities (Hogberg, 2021; Rudolph et al., 2021). The organizational processes following COVID-19 refer to emergent changes in work practices such as working from home and virtual teamwork (Dingel and Neiman, 2020) and emergent changes for workers in response to the adverse effects of the pandemic such as social distancing, stress, and unemployment (Kniffin et al., 2021).

Following the recommendations from Wu et al. (2021), more research is needed to understand such organizational processes and how leaders engage in emotion management and regulation. According to Wu et al. (2021), effective crisis leadership rests on the ability to understand and attend to employees’ reactions and regulate one’s own emotions to instill a resilient response despite extreme contextual challenges. Specifically, there is a need for research to address how leaders can mitigate both negative emotions and hardships, and how they can elicit positive emotions, promote functional coping, and resilience following crisis situations (Wu et al., 2021).

Emerging research from Taiwan suggests that authentic leadership positively affects social exchange relationships and trust, whereas social exchange relationships positively affect trust after COVID-19 (Chen and Sriphon, 2022). Moreover, they found that a social exchange relationship had a mediating effect between authentic leadership and trust (Chen and Sriphon, 2022). In the same vein, a study of crisis leadership in Norwegian school principals corroborates the significance of attending to social exchange relationships and emotion management processes. One of the main findings from this study was the need for the school principals to continuously adapt to a complex and evolving pandemic, while attending to the needs of teachers, families, and students (Lien et al., 2022). The significance of attending to emotion management and a caretaker leadership is also emphasized in a study of crisis leadership practices in rural school principals in the U.S. (Hayes et al., 2021). Furthermore, a study from small business ventures in Portugal in the first phase of the pandemic utilized a case study format and a sensemaking perspective to examine how senior managers coped with the challenges from the pandemic. This study underscores the significance of engaging workers in cognitive shifts followed by shifts in practice to cope, adapt and to offer swift and effective solutions to specific problems, making the most out of existing skills and resources (Sarkar and Clegg, 2021). Taken together, the emerging research on crisis leadership during the pandemic emphasize how successful crisis leadership processes involves sensemaking (Combe and Carrington, 2015), attending to social exchange relationships, and maintaining a proactive approach to cope with the extreme context of the pandemic.

To this end the Cognitive Activation Theory of Stress (CATS: Ursin and Eriksen, 2010) presents a useful perspective on how business leaders interpret and cope with the emotional and cognitive aspects of COVID-19. The CATS framework defines coping as a positive outcome expectancy, which means that the leader expects that his/her response to the situation will provide positive outcomes for the employees and the organization. In response to the extreme context and potentially adverse effects from the pandemic an authentic leader who engages in social exchange relationships by attending to the emotional stress of employees may be in a better position to overcome fear and rumination in support of a productive and resilient response to the extreme context of the pandemic (Sarkar and Clegg, 2021; Chen and Sriphon, 2022; Lien et al., 2022).

Taken together, the extreme context of COVID-19 presents an opportunity to respond to the call for more research on emotional aspects of crisis leadership (Wu et al., 2021), by examining cognitive expectation, social exchange relationships, and sensemaking processes in a group of Norwegian business leaders in the early stage of the pandemic. Following from in-depth interviews an objective is to gain knowledge on how the business leaders managed negative emotions (e.g., anger, anxiety, fear) as well as how they instilled positive emotions and resilience in their followers during the pandemic.

## Materials and methods

The study adopted a qualitative method in the form of semi structured interviews using a phenomenological hermeneutic design that aims to derive knowledge regarding how the pandemic influence the crisis leadership of Norwegian business leaders during the COVID-19 pandemic (Creswell et al., 2018). A qualitative approach was preferred in part because crises have sudden, intense—yet sometimes short-lived—outcomes that are processed through sensemaking, construction of meaning, and tensions experienced by individuals (Combe and Carrington, 2015). These psychological processes and behaviors are often difficult to fully capture using quantitative methods. Therefore, a qualitative study was preferred to obtain a deeper understanding of the phenomenon of leadership in times of crisis, rather than generalizing findings across contexts and samples. Thus, the outcome of the research process rests on an iterative process between the business leaders expressed experiences and the researchers’ interpretations. To clarify and disclose our prior understanding, a brief research proposal was submitted to the Regional Ethics Committee as part of the approval of the study (REK, no. 187331/2020).

### Recruitment and sample

A heterogeneous purposive sample of leaders from private sector organizations in Western Norway were recruited. The main sector branches, including finance, consultancy, denominations, trade, building and construction, IT, and industry. About 8 months into the pandemic a diverse sample of business leaders were approached by telephone or e-mail and encouraged to take part in the study. Only one of the subjects declined to participate, for the most part due to personal reasons. Thus, informed consent was obtained from 11 candidates who agreed to take part in the study. Of these, seven informants were males, and four females. Experience in the leadership role ranged from 1 to 25 years. All candidates had a senior management position when the pandemic emerged (March 2020), and still had the same leadership position at the time of the interview (November 2020). Thus, the sample represented a diverse group of business leaders who were actively involved in managing the pandemic and could present a rich and diverse personal perspective on the situation.

### Data collection

Due to the ongoing COVID-19 situation the interviews were conducted *via* Teams during a 6-week period. The interviews were based on a semi structured format with open-ended questions. Sample questions are shown in Table 1, and focused on the leaders’ experience of challenges, changes in the business context and crucial decision during the first 9 months, including two waves of the pandemic in Norway. Follow up questions tapped into cognitive and emotional aspects of the pandemic, such as perceived changes in work-home balance, interpersonal relations, trust, emotions, or changes in leadership style.

**TABLE 1:** Core topics and sample questions from the interviews.

Most significant experiences	**What do you see as the most significant experiences you have had as a leader since 12th of March?** •Have these experiences changed you as a leader? •Have you had doubts or felt insecure? If so, how did you manage?
Most significant challenges	**What have been your biggest challenges during this period?** What makes you mention this? •What made you come up with this as a solution? •Why did you choose to solve your challenges in this way?
Changes at work	**Have you approached work issues in a different way during the pandemic?** •Has the pandemic led to changes you have not seen or believed could be possible before? •If so; what do you think was the reason it was possible now? •Has the pandemic opened any new opportunities?
Interpersonal aspects	**What is your experience concerning the work-home balance during the pandemic?** •Has it, at any point, been difficult to trust the employees’; effort during home-office? •Have you experienced any changes in your relations with your colleagues during the pandemic?
Personal experiences	**Do you think your personal qualifications have helped you during the pandemic?** •Anything that has made your role as a leader easier? •Have you received personal feedback on you as a leader during the pandemic?
Emotional aspects	**When you think back on the last 8 months during the pandemic; what emotions comes to mind?** •Do you see any emotional aspects of leadership in a different way now than before?

Each interview lasted between 55 and 110 min. For reasons of privacy and anonymity, audio, but not video was recorded. A pilot interview was undertaken, to secure that the interview format and technical processes were feasible. All recordings were transcribed verbatim immediately after they had been completed. VDO, JS and SHRS performed the interviews. The transcripts were shared and discussed with peers after each interview. After eight interviews, a saturation was starting to form. Attending to this, the next three interviews were undertaken with an extra effort to elaborate on discrepancies and contribute to thematic clarifications. However, since these last three interviews provided limited new perspectives, the information power of the sample were concluded to be sufficient to conduct a responsible analysis (Malterud et al., 2016). Theory was used to sharpen the focus for interpretation and discussions (Malterud, 2001, 2016).

### Analysis

Systematic text condensation (STC), a descriptive cross-case analysis strategy, was used for analysis of the transcripts (Malterud, 2012). In accordance with STC the transcripts were analyzed in four steps: (1)All the transcribed interviews were read to obtain a general impression of the material and to identify five to eight preliminary themes. (2) A second carefully reading of the manuscript were then performed to develop code groups based on the preliminary themes and identify units of meaning related to the code groups. (3) By distancing from the units and focusing on the phenomena emerging, these codes were then condensed into groups and subgroups. The analysis program NVivo (Version 12) was used for the analysis (QSR International Pty Ltd., 2018). Key quotes to illuminate the main results in each subcategory were identified in this step. (4) The fourth step synthesized the contents of each code group to present a reconceptualized description of each category concerning coping strategies and adaptive approaches in private sector leaders in Norway during the first wave of the COVID-19 pandemic in Norway. Table 2 provides an overview of the analytical process of the study.

**TABLE 2:** An overview of the analytical process of the study.

Themes	Second order constructs	First order codes-data exemplars
A surreal feeling	State of emergency at the beginning	*“Right, so in the period where they closed the schools, I am married to a man who can’t work at home, right, he is a contractor, so he’s always out, so I sat at home with three children, had more than ever at work. I felt like I was working 24 h a day in the first period, it’s like I have displaced it, like a birth. A corona-birth.”*
	The leadership role in a state of emergency	*“Hence the focus in the beginning was call it emergency leadership in the organization, preparedness in relation to being close, so that we can adjust if necessary, and partial control so that we have to know, do we have enough work for everyone? Are we working toward companies that we think are going out of business now? Do we get paid in relation to the assignments we have delivered? (…) A national and regional emergency group was established.”*
	Adapting over time to a new normal	*“I don’t think things will go back to normal. I believe this represents a shift.”*
Caring for people	To lead with human compassion	*“we as leaders*…*Pandemic means to take two steps forward, it means to be clear, It means we (leaders) can’t hide. We have to step up, we have to understand how people are, we have to understand the risks. AND we have to try to give as much security and predictability as we can. We have to show that we care.*
	To fill the need for belonging when one cannot meet	*“And because there are so many employees who work hard, who contribute and create things, we have so much. There is an incredible focus on social activities at work. Just putting off on hour for Christmas lunch, and have food delivered on the door, with activities on Zoom and breakout-rooms, and you name it. Even had a Christmas-gift delivered by post.*
Cohesion	Cooperation	*“I haven’t thought about it before. You’re onto something essential there, because it’s weird, but I think we’re even closer now. One should think the opposite when the employees are let off, right.”*
	Few conflicts despite major changes	*“Because everyone understands how critical it is, and understand that if this turns really bad, we won’t even exist. We’ll have to find new jobs, and the seriousness of that is much larger than we have ever experienced before.”*
Resilience	Experiencing stress and stress management	*“I have to dare to take chances on things I don’t know the answer to.”*
	Personal resources	*“On a professional level, I am triggered by this, to be honest (laughs).”*

## Results

The analysis revealed several aspects related to how the pandemic had influence the crisis leadership of Norwegian business leaders during the early stage of the pandemic. For many, the pandemic caused a high rate of quick, unplanned changes. Some were implemented overnight. Many had been embossed with constant changes in infection control rules, digitalization, concerns for the employees, income, and operation challenges. Throughout, the leaders described a situation where they had to face insecurity, frustration, and challenges, but also had experienced profoundly gratifying moments.

A surprising finding was that the leaders described overwhelmingly more positive than negative experiences despite the uncertainties and protracted hassles from the pandemic. Several of the leaders had received feedback from their employees indicating that they had handled the situation well, attended to their needs and provided them with timely and relevant information. Some even indicated a growth in employee satisfaction and expressed pride and gratitude over their accomplishments. One leader expressed this notion in the following way:

*“But to me, there have been a lot of positive changes. Many good things to bring along further. Even though the situation of course deep down has been challenging, and thus negatively charged.”*

### Surreal

Most informants remembered the time around 12th March as a significant date that signaled significant change, for example when the production facility had to be stopped overnight, and how the production facility normally bustling with people turned cold and empty. For many, the weeks leading up to 12th March had been marked by uncertainty and challenges. One of the leaders elaborated on this moment where she realized the scope of the situation:

*“For such a state of emergency and crisis, we cannot travel, cannot this and that. You get a lot of restrictions, right. There are many [restrictions], right, like it was during the second world war. You can’t walk, you can’t move, you cannot even walk outside your door, right? And there is danger. And there is, after all, threat of life, and insecurity, and. And my God, the pictures from Italy. That’s how it was in the beginning. It was a crisis.”*

Although similar notions were shared by most leaders, a few did not refer to the pandemic in the same negative sense. One of the leaders agreed that the pandemic had offered challenges but did not see it as a crisis. Another described that he was used to large fluctuations and uncertainty in the markets, so the pandemic mirrored earlier experiences. A third leader simply stated that the pandemic hadn’t presented too many challenges.

Most of the leaders alluded to the impression that the start of the pandemic was marked by mobilization and that many significant decisions had to be made in rapid succession. At the same time, some mentioned that they held back some difficult decisions to see if more information would become available and thus provide a better basis for the final decision, instead of reacting too quickly. Some pointed out that they trusted their gut feeling. An experienced leader described this perspective as follows:

*“In a situation like this, my experience is that it requires clearer leadership. It requires clearer, that is, you do not ask for emergency preparedness, but I think elements from there, in my leadership, because in emergency preparedness you must be so much clearer. If there is a crisis, then there must be clarity, and you must provide security, and I will take some of that with me now, in what we do.”*

Many of the informants described how they rapidly had to implement multiple significant changes within a short timeframe. Within 1 week, infection control routines, home offices, daily emergency morning meetings were established, and several had an increased focus on control and reporting. Yet, in many businesses the pandemic quickly became the new normal, it became part of the routine; “*In the beginning, we called this emergency meetings, but now we do not overdramatize it anymore.”*

Although the leaders quickly seemed to adapt to the new situation, some still suggested that they perhaps had not quite been able to absorb all the changes and disruptions. They realized that the pandemic had an emotional and personal impact. When asked about the personal aspects of the situation, one of the leaders said; *“It’s funny you should ask, because yesterday I said to my wife; “I wonder when I will have a reaction to this.”* Another leader got an emotional reaction during the interview and started to cry, when asked about the personal side of the situation:

*“This [interview] has been like a therapy session, to me. Like, I don’t think I have systematically, over such a long period of time, thought about the whole situation, so the interview and your questions and the process you’ve taken me through, well it probably started something I haven’t taken time to think about before*…*..”*

### Caring

Several of the leaders said they had become more sensitized to their own and their coworkers’ values and priorities. One of the informants stated that he saw no contradiction between being an empathic leader and caring for the employees, while at the same time managing a profitable business enterprise. In the same vein, some leaders expressed that they were working for their employees, their business concept was based on the success of the individual contractor, thus caring for the employees was crucial to the business. By caring for the employees, leaders had found a way to instill trust in the leader—member exchange. One put it this way:

*“My experience is, if you have trust, you get a larger room of maneuver in a situation like this, than if. you have to build trust, trust must be present, they have to trust you as a leader, that you make the right. choices, right? Well, if you lose the trust, they will not listen to you. If you’re able to keep the trust, the room of maneuver is larger. Everybody understands that someone’s got to make the calls, right.”*

Several of the informants expressed a lot of empathy and concern for their coworkers and employees. They felt a need to follow up closely and support their needs in a way they had not seen necessary before. Some were especially worried about their young and single employees that most likely lived alone, thus did not have access to the same social support as coworkers with families. Several leaders were mindful about keeping closer contact with the employees and to listen in on how they were doing. Many were consciously reserving time in digital meetings for “coffee talk” and encouraging employees to take necessary precautions to self-care during these very special times. One of the leaders referred to this situation as a human crisis:

*“Because it is something about humanity in a crisis, a human crisis, I think. Then you just must step down and be a human being, right. So, in that email I remember writing to the employees, right, it must have been something like I talked about how I had been sitting and doing x amount of work meetings and closed down this and that, the other stuff like reports and such, meanwhile [I was] writing about dinosaurs in English and something else, because it was home school, right.”*

Several of the leaders said they had focused more on the need for safety for their employees during the pandemic. One way to accomplish this had been to involve them more in the decision making. One informant said:

*“I think leadership is about people. So, it’s not about an excel sheet. I think there are many who miss this. So, during these times you can put everything in a column in an excel sheet and you also get a number at the bottom, and you also decide on it. I think that’s bad.—Bad! It is part of the decision basis, but it cannot stand alone. So, I’m joking about this. There are far too many “white collars” in the company management in many places. I think the best leaders are those who care and who are concerned with the whole.”*

The disruptive effects of the pandemic soon fueled the issue of business closures and the need to lay off staff from work. These decisions were not easy, and our informants emphasized the need for thorough communication about the situation, and how the leaders worked to keep the workers in the loop about what was happening with the business after the layoff. All the leaders had decided to bring back the workers who had been laid off as soon as possible, one of them even before getting the approval signal from the top leaders.

In two of the organizations, the leaders and business partners decided together that they would take the financial risk and not lay off workers because they could afford it. They chose to not extract the financial dividend and instead used the organization’s equity to keep people at work, despite low potential income and the legal option to lay off people from work. One experienced leader described how she even went against her international leadership, because they wanted her to lay off employees, while she was not willing to. She described a process in which she and her board of local leaders decided not to lay off people:

*“and when that landed in me, that we would not do it before it was necessary, it was so, it landed so steadily in me, that I just felt that I was not going to move an inch (.). Then they must. then they must. then they must order it, right, done, in a way (.) because we are a company that has done well, and has done well for many years, and the employees are the whole. They are the reason why we do well. And suddenly to act surprisingly fast in such a situation. That it’s them. That the first thing you do is sort of just to. No, it was just very, very wrong. And then I stood firm, and I stood in the pressure, and it was persistent for a while. None could make me go away from that [decision].”*

### Cohesion

Several of the informants said they had experienced a stronger unity and cooperation in the company during the pandemic. Some had noted more cooperation between departments, other mentioned improved relations in the management team, between different areas of activity, or improved relations with different other companies. Three of the informants used the term “common external enemy” to describe the pandemic and other leaders mentioned the shared experience of an external threat that set the premises for daily operations and any changes that needed to be introduced. Several stated that they experienced being closer to each other in the company, and had greater transparency in the business, due to an increased number of meetings, to alter the flow of information. Some also pointed to this as a success factor during the pandemic and believed they would continue like this even after the pandemic, since it had such a positive effect for the business. One of the leaders put it this way:

*“Transparency has increased. Because we meet, everyone is up to date, at least roughly, on what the other areas of activity must do. What their resource situation is. So, there are in a way many positive effects of that. So, you can say, we had to lead differently during the pandemic than we did before. But we*…—*we will not lead differently after the pandemic compared to what we did during the pandemic if you understand. For being so close, we see it has been a success factor.”*

One manager pointed out how lack of information can lead to a breeding ground for suspicion and mistrust and can develop into a problem orientation among employees. Some of the informants pointed out that it seemed that there was a greater understanding and awareness among the employees, and that they had a desire to help each other:

*“And then I feel that this crisis has moved our company in the right direction related to collaboration, and generosity and coexistence and cooperation, and that is something that means a lot to me, and that I am very passionate about, and then I think in fact, we have received some help along the way, for there are many who have opened their eyes to that “hm, if we have it as our main rule instead of sitting and grumbling about things and holding on to things and saying that this is mine, then it will be much more fun for everyone.”*

Some believed that there would have been far greater friction and more conflicts if these changes came from within, and not from without, in the form of the pandemic. Some experienced that the willingness to change had been greater due to the common understanding that the pandemic was an external threat. A leader described this as follows:

*“It would have been terribly much more demanding, like, you would have had a lot more discussions, like, you have team players and then you have opponents in groups, which you as a direct leader must deal with. I believe that we, that we, all see what we have managed now, I see the willingness to change is present in far more people than we have had before. They have seen that, they have experienced for themselves that we change for the better, not for something worse, we have changed for something that actually works, given the circumstances, then.”*

Several informants pointed out that many employees were starting to get fed up, tired of home office and the fact that they were not allowed to meet colleagues, but they still experienced an enormous understanding of the infection control measures presented by the government. Some of the informants also pointed out how the authorities had led during the COVID-19 pandemic and believed that the public narrative had made it easy to follow up and had contributed to a sense of unity and security. The increased digital interaction and loss of contact with colleagues due to infection control measures, were only mentioned as conflict-escalating by one leader. In a business that had a latent conflict before the pandemic, she experienced it as necessary to be physically present and this was much more difficult to follow up during the pandemic.

### Resilience

Many of the leaders said they were used to stressful workdays with many deadlines, long working hours and a blurred line between work and life. So, most of the informants described that they were coping quite well with the pandemic and not having a problem with the new work routines. Some highlighted how they keep calm in stressful situations. Several were excited, or triggered, to be faced with a demanding and stressful situation like the pandemic. One informant described it like this:

*“I find it fun. I like a bit of action (….). Although this is a bit risky to say, because it is, people are dying, it’s horrible, but when it comes to emergency preparedness, it triggers me.”*

Several informants talked about how the lines between work and family life had become increasingly blurred during the pandemic, to the point of non-existence. Some explained it being due to working from home office. Another mentioned the absence of social activities, it was just easy to continue working:

*“So (back) then, you just folded the computer down and put it in your bag again, but now, if you take the computer away, the screen, the headset, and the mouse and all kinds of wires are still there just lying around. You don’t close down in the same way.”*

Despite these comments, most of the leaders managed to strike a balance between work or leisure time and did not have difficulty taking breaks from work. In between work periods the leaders took time off by working out, played golf, cooked, went for walks, traveled to their cabin, drove their motorbike, or spent time with family and friends.

*“You don’t leave it when you go home. You bring it with you all the time, and you must work a little in the evenings and such. But I don’t feel like, it hasn’t been, a problem for me. But I hear many other people saying it has been a problem, and I understand that. So, you have to kind of be a little structured, and to have a little discipline to take time off.”*

Several leaders said they are normally quite carefree and optimistic by nature, confident that things will work themselves out. They were looking forward and expressed confidence in their ability to cope with the future. They appreciated this opportunity to make a difference in a difficult situation. One leader said she slept well during the nights and was not angry or worried. Another said she was adaptable and flexible, although many also found it difficult to highlight their personal achievements during the pandemic and wanted to downplay their role since it felt like being “*a little braggy.*” In discussing their personal take on the pandemic situation, one of the leaders referred to a well-known Scandinavian heroine, Pippi Longstocking, in describing her way of thinking like this: “*As Pippi says:” I have never done this before. I am sure I will do great*!”

## Discussion

**FIGURE 1:**
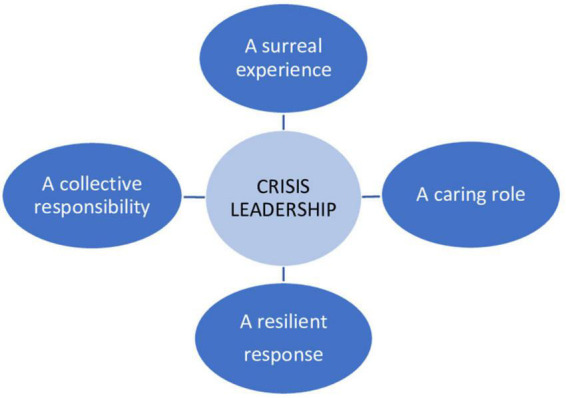
Factors relevant for crisis leadership experienced by Norwegian business leaders in the early stage of the pandemic.

The aim of the present study was to explore crisis leadership in Norwegian business leaders during the early stage of the pandemic. A special emphasis was placed on how cognitive expectations, social exchange relationships, and sensemaking processes were embedded in the crisis’s response from the business leaders. The analyses demonstrated four factors relevant for crisis leadership in the early stage of the pandemic. The informants talked about a surreal experience, empathy and caring for the employees, and a strong collective responsibility and unity in the organization. At the same time, they all expect to handle the situation well. Below we discuss the impact of these findings (for an overview of the themes, see Figure 1).

### A surreal experience

COVID-19 represented a surreal and extreme context to most of business leaders. They expressed a sense of severe emotional distress and likened the pandemic to a war like crisis with potentially severe implications for individuals and the organization (Dionne et al., 2018). The pandemic was seen as a cascading event that affected almost every aspect of human life, including work life, private life, recreational and social opportunities, travel, and personal physical and mental health, to name a few. The pandemic emerged as a protracted crisis where uncertainty, insecurity, and frustration were salient features likening the pandemic to other man-made or natural disasters (Eid et al., 2005). Adding to the uncertainties and challenges were the fact that only one of the businesses had a contingency plan for this type of cascading and protracted event, leaving most of the leaders with limited guidance and few resources to mitigate the intensity of the crisis (Hannah et al., 2009). Even the only business with a contingency plan, did not have effective infection control measures in place until the week before Norway was shut down. With little, if any training and preparations the business leaders had to serve as crisis leaders as the pandemic unfolded (Bundy et al., 2017). Thus, most of the business leaders were unprepared and had to assess and respond to the situation as it unfolded. Confronted with the extreme situation, the narratives from the business leaders underscores the significance of the emotional aspects of crisis leadership (Wu et al., 2021) and how they struggled to make sense and find their way forward. The leaders descriptions from the first weeks of the pandemic as a surreal war-like experience, presents a personal narrative of the business leaders as warriors, resembling military leaders in extremis situations (Hannah et al., 2009). Coming to terms with their fear and the grim realities of the situation emerged as an important to aspect of their sensemaking process, “*And there is danger. And there is, after all, threat of life, and insecurity, and. And my God, the pictures from Italy. That’s how it was in the beginning. It was a crisis.”* Seeing the pandemic as a threat, not only to their own business, but also as a crisis for the society at large told the business leaders that they were not alone in confronting the situation (Garretsen et al., 2022). These noticing and meaning-making facets in the early period of the pandemic served an important role in justifying the subsequent action facet of the sensemaking process (Maitlis and Christianson, 2014), and in shaping the business leader’s role and subsequent behavior. Although the pandemic was unexpected and came without a business playbook, the leaders followed a basic strategy of attending to day-to day problems and caring for their employees.

### A caring role

Confronted with the dire consequences from COVID-19, the situation presented an opportunity for the business leaders to express their values and way forward by renewing and invigorating their relationship with the employees. Expressions like, *“leadership is about people, not excel”* comes across as a clear commitment to attend to the needs of the employees and care for their needs (Arnetz, 2005). Adding to this, the business leaders underlined how they had worked hard to increase their communication and daily information, to create a sense of structure, ease the unrest, and give the employees a sense of stability. The business leaders detailed how they had restructured organizational processes, implemented new routines, attended to customers and clients, but first and foremost instilled hope, trust, and resilience in their employees. The focus on hope and trust reflects a mindset among the leaders that includes positive response outcome expectancy that according to the CATS (Arnetz, 2005; Ursin and Eriksen, 2010) will contribute to resilient employees and sustainable organizations through healthy and adaptive stress responses. The most experienced leader in the group emphasized how she believed in clarity and the importance of providing a sense of security and coherence to the information she disseminated to her employees, “*If there is a crisis, then there must be clarity, and you must provide security.”* Thus, the meaning-making aspects of crisis leadership and a positive response outcome expectancy emerged as two important aspects of their crisis leadership.

The need to establish a comprehensive and shared understanding of the situation based on observed data and facts became an important aspect of crisis leadership (Weick et al., 2005). In the pandemic, people had to make sense of large amounts of often conflicting information and often had to attend to competing demands for attention (Christianson and Barton, 2021). A shared understanding of the situation was an important part of establishing a trusting relationship between leaders and employees that also gave legitimacy to the difficult decisions that had to be made; *“My experience is, if you have trust, you get a larger room of maneuver in a situation like this, [….] If you’re able to keep the trust, the room of maneuver is larger. Everybody understands that someone’s got to make the calls, right.”*

### A collective responsibility

The business leaders were forced to implement a host of radical changes over a short time. Organizational restructuring is never easy and can ignite conflicts and emotional stress. Faced with the hardships of the pandemic, structural changes soon became inevitable. To this end the investment in establishing a shared understanding of the situation not only contributed to trust, but also nurtured a co-evolutionary framework of organizational sensemaking (Christofaro, 2022), that apparently also had a significant impact on emotional schemata in collective sensemaking processes. Paraphrasing one of the business leaders; “*Never underestimate the effect of a common external enemy”* when difficult decisions had to be made.

Several of the business leaders were forced to lay off people or experienced a dire financial situation due to the disruptive effects of the pandemic. The conflicting issues of caring for their employees in troubled times, and coping with the financial fallout from the pandemic, soon emerged as a significant dilemma. Two leaders extracted the financial dividend to keep people at work, although they were falling short on incoming orders. Another leader even went against their international leadership policy in laying off people, arguing that “*the people are the organization, and the whole reason we do so well.*” But, for those who had to lay off people, the leaders emphasized communication, keeping them in the loop and following up to have them back as soon as possible. The hardships from the pandemic seemed to have strengthened unit cohesion (Bartone et al., 2002) and contributed to unity and acceptance of needed business restructuring during the crisis. To this end, a vital element seems to have been the efforts to establish a shared understanding of the situation (Christianson and Barton, 2021) and to swiftly establish trust in the leaders (Hyllengren et al., 2011).

Collective sensemaking is a significant element in collective transformations (Weick et al., 2005; Stephens et al., 2020) that challenges old interpretations and require people to craft new understandings in response to emerging problems. All the business leaders in this study were facing the complex and ambiguous aspects of the pandemic within the framework of the Norwegian legal and political system. Under the slogan “*working together*,” the Norwegian government invested heavily in collective meaning-making. Regularly ministers and senior government officials communicated to the public to build a supportive and cohesive national culture to encourage compliance with the preventive measures to curb the spread of the virus. This, strategy seemed to pay off and public trust in government increased significantly from March 2020 in the early stages of the pandemic (Christensen and Laegreid, 2020). Thus, the local, organizational, and national efforts were quite aligned in establishing a shared narrative of a sustained and collective effort to fight the pandemic.

### A resilient response

From the interviews, it became clear that the business leaders differentiated between the first weeks when the pandemic hit, and their experience of the current situation 7–8 months into the pandemic. Expressions like, “*In the beginning, we called this emergency meetings, but now we do not overdramatize it anymore”* reflects how the leaders seem to have adapted and were coping with the current situation, expressing habitual processes in the same ways as trauma survivors (Johnsen et al., 2002). Still, the pandemic was ongoing and not all the leaders had been able to process and come to terms with the emotional aspects of their situation. For some, the interview situation emerged as the first opportunity to sit back and reflect. Like one of the informants said, “*the interview and your questions [*……*] probably started something I haven’t taken time to think about before.”* A compelling observation seems to be that the pandemic also emerged as a transformative moment, not only for the organizations and the employees, but also for the business leaders themselves. The accounts from the business leaders in the present study aligns well with the sense of personal mastery and resilient response from a group of Norwegian school principals who were confronting COVID-19 in the same period (Lien et al., 2022).

The results from this study also show how a crisis can give rise to new opportunities (James et al., 2011). Despite a negatively charged situation, the business leaders pointed to several positive outcomes from the pandemic. Some even liked the action and felt energized from the challenges and alluded to a readiness for organizational change (Armenakis et al., 1993). Several expressed positive expectancies from the outcomes and future of the business after the pandemic. A quote like “*This I’ve never done before. I’m sure I will do great*” resonates well with CATS (Ursin and Eriksen, 2010) in upholding an optimistic and resilient take on the situation and their ability to cope with organizational stress (Arnetz, 2005). A positive response outcome expectancy will in contrast to none or a negative outcome expectancy, lead to a healthy adaptive stress response, and growth when faced with threatening events (Ursin and Eriksen, 2010). Positive expectations may also engender better social relations and positive emotions providing support during times of difficulty (Taylor et al., 2000). The result from the present study corroborates and extends other studies in suggesting that the pandemic have provided an opportunity to explore new opportunities and changed work related practices (Hogberg, 2021; Rudolph et al., 2021). The perceived support, cohesion, and trust could provide a safe opportunity to engage in new work routines and practices such as working from home or engaging in virtual teamwork (Dingel and Neiman, 2020). In the same vein, these same factors could also be seen as important aspects of crisis leadership to fend off or reduce the potential adverse emotional or psychosocial effects of the pandemic in the form of social distancing, alienation, stress, and unemployment (Kniffin et al., 2021).

### Study limitations

Some limitations should be observed when interpreting the results of this study. First, the sample of business leaders are relatively small, and the exploratory nature of the study present causal attribution. Still, the interview sessions were extensive and provided a rich material, on leadership challenges from different small, medium, and large business enterprises that can inform future research. Second, the interviews were conducted *via* Zoom or Teams and not in a live, physical situation. Although, the leaders and interviewers were quite used to this format and it made scheduling of appointments more flexible, the lack of physical proximity may have made it more difficult to assess and attend to visual cues or body language. Third, the informants were asked to recall back 6–7 months in time, and this retrospective approach is vulnerable to the constructive nature of memory when it comes to emotional aspects (Kobbeltved et al., 2005). Nevertheless, the protracted and emotionally charged nature of the pandemic implicated that all the informants still were actively involved in dealing the pandemic situation at the time of their interviews and in the interviews presented a vivid and true to life account of their experiences (Talmi, 2013).

## Conclusion and future direction

The present study addresses important issues regarding how business leaders rapidly had to shift from risk management to operational resilience management (Pescaroli and Needham-Bennett, 2021). To our knowledge, this exploratory study provides the first in-depth assessment of how Norwegian business leaders experienced and coped with the disruptive effects of COVID-19. The sudden onset, protracted nature, and the severe consequences of the crisis posed a significant threat to business routines and prompted a need for crisis leadership. From a sensemaking perspective, the business leaders attend to the noticing, meaning making, and action facets of sensemaking to build trust, care for the employees, and instill needed actions to adapt to the extreme context of the pandemic (Christianson and Barton, 2021). Taken together, the present study adds to our understanding of how sensemaking processes were embedded in- and contributed to social exchange relationships between the business leaders and their employees. Furthermore, the study also illustrates how effective crisis leadership rested on cognitive appraisal processes that instill positive response outcome expectancies, optimism, and resilient responses to the disruptive effects of the pandemic.

The present study provides multiple opportunities for future research. One possible avenue could be to conduct a more extensive survey on lessons learned, including similarities and differences in how public and private sector leaders have managed the pandemic. From a mixed methods perspective (Schoonenboom and Johnson, 2017) a combination of elements from qualitative and quantitative research approaches could be applied for the broad purposes of breadth and depth of understanding and capturing the essential aspects of crisis leadership during the pandemic. A timely issue in future research could be to apply a mixed methods approach to examine to what extent lessons learned from crisis leadership in the pandemic are applied after the pandemic. Such a study should not only focus on the leaders, but also include the employees and organizational perspective. Finally, future quantitative studies should investigate the relationships between the four factors identified to be of importance for crisis leadership: the experience of a surreal world, caring of employees, a collective responsibility and resilience.

## Data availability statement

The raw data supporting the conclusions of this article will be made available by the authors, without undue reservation.

## Ethics statement

The studies involving human participants were reviewed and approved by the Regional Committee for Medical and Health Research Ethics (REK) West (ref. no. 187331). The patients/participants provided their written informed consent to participate in this study.

## Author contributions

VDO, JS, SHRS, AH, and JE together conceptualized the article and together edited and finalized the article. VDO, JS, and SHRS conducted the interviews and the analysis. VDO wrote the first version of the manuscript as described in the manuscript. All authors approved the submitted version and can be held accountable for the content.
